# Chemical Composition of Tobacco Seed Oils and Their Antioxidant, Anti-Inflammatory, and Whitening Activities

**DOI:** 10.3390/molecules27238516

**Published:** 2022-12-03

**Authors:** Ji Gu, Xiaoyu Zhang, Biqing Song, Dongjie Zhou, Yongzhi Niu, Guiguang Cheng, Yunye Zheng, Yudan Wang

**Affiliations:** 1 Yuxi Zhongyan Tobacco Seed Co., Ltd., Yuxi 653100, China; 2 Faculty of Food Science and Engineering, Kunming University of Science and Technology, Kunming 650500, China; 3 Key Laboratory of Chemistry in Ethnic Medicinal Resources, State Ethnic Affairs Commission and Ministry of Education, Yunnan Minzu University, Kunming 650500, China

**Keywords:** tobacco seed oils, fatty acids, antioxidant capacity, cytoprotective effect, tyrosinase

## Abstract

Tobacco seeds are a valuable food oil resource, and tobacco seed oil is rich in nutrients, especially polyunsaturated fatty acids. The aim of this work was to perform a comprehensive study on the chemical constituents, and the antioxidant, anti-inflammatory, and whitening activities of tobacco seed oils (NC89 and BS4). A GC/MS analysis revealed that NC89 and BS4 had 11 and 6 volatile compounds, respectively. The PUFA contents in NC89 and BS4 were 74.98% and 72.84%, respectively. These two tobacco seed oils also presented good radical scavenging capacities with the neutralization of ABTS, OH^−^, and superoxide (O_2_^−^) radicals in a concentration-dependent manner. Meanwhile, NC89 and BS4 inhibited reactive oxygen species (ROS) accumulation and cell apoptosis, enhanced SOD and CAT activities, and increased the GSH content in H_2_O_2_-induced HepG2 cells. In addition, NC89 and BS4 exhibited significant anti-inflammatory activities by inhibiting the expressions of NO, TNF-α, IL-1*β*, and IL-6 in LPS-induced RAW.264.7 cells through the regulation of the MAPK signaling pathway. Moreover, NC89 and BS4 expressed whitening activities by inhibiting tyrosinase activity and intracellular melanin production. Therefore, tobacco seed oils could be used as an important oil resource for the development of high value-added products.

## 1. Introduction

Tobacco plant (*Nicotiana tabacum* L.) is an important commercial crop that is widely cultivated around the world [1,2]. Its leaves are generally processed into various tobacco products including cigars, bidis, snuffs, hookah, and gutka. However, tobacco seeds are wasted byproducts of commercial leaf production. Recently, tobacco seeds have been developed as biodiesel, animal feed, paint, soap, and seed oil [3]. A study showed that tobacco seeds have oil contents of about 30–40%, which is higher than that of some other oil crops, including soybean, cotton, and olive [4]. Tobacco seed oil contains a variety of beneficial substances, such as triacylglycerols, phospholipids, tocopherols, sterols, and unsaturated fatty acids [5]. In addition, the major unsaturated fatty acids in tobacco seed oils are linoleic acid, oleic acid, palmitic acid, and stearic acid [6]. Unsaturated fatty acids have the potential for treating cardiovascular and cerebrovascular diseases [7]. Thus, there is an urgent need to perform a further study on the chemical composition and biological activity of tobacco seed oils for commercial application.

Reactive oxygen species (ROS) are important signaling molecules in the regulation of cell survival and cell death. However, if excessive ROS can’t be scavenged in a timely manner, oxidative stress can occur, which can cause irreversible oxidative damage to the lipids, proteins, and DNA. Oxidative stress is closely related to inflammatory response, which can induce a series of chronic diseases. It has been reported that inflammatory cytokines will influence melanocytes’ immune and metabolic functions, contributing to melanin deposition in the skin [8]. Hyperpigmentation in the skin, the suppression of oxidative stress, and inflammatory response is a potential strategy for inhibiting the deposition of skin melanin [9]. In addition, a study has reported that some plant oil extracts (olive oil, olive pomace oil, sunflower seed oil, etc.) exhibited significant antioxidant and anti-inflammatory effects [10]. However, the studies about the antioxidant and anti-inflammatory abilities of tobacco seed oils are limited. Therefore, it is important to explore the antioxidant and anti-inflammatory abilities of tobacco seed oils for skin whitening via reducing melanin accumulation.

In China, Yunnan province is the most important region for tobacco cultivation. Tobacco seed is a byproduct of tobacco leaf production and is traditionally processed as an edible oil by the local people. Although the tobacco seeds are very small in size, their biomass per plant is very large. For tobacco cultivation, the NC89 and BS4 tobacco species have a higher planting density than other species, thereby leading to a large amount of NC89 and BS4 tobacco seeds. However, the chemical composition and biological activities of the oils from the NC89 and BS4 tobacco seeds have not been studied. In order to increase their economic value, the chemical composition, and the antioxidant, anti-inflammatory, and whitening activities of tobacco seed oils (NC89 and BS4) were explored in this work. The fatty acid composition and chemical constituents were analyzed by gas chromatography coupled with FID or MS detectors. The antioxidant activities of the NC89 and BS4 oils were determined by scavenging ABTS, OH^−^, and O_2_^−^ radical assays and inhibiting the ROS production in H_2_O_2_-stimulated HepG2 cells. The anti-inflammatory effects were determined by measuring the expressions of NO, TNF-α, IL-1*β*, and IL-6 against LPS-induced RAW264.7 cells. Moreover, the whitening activity was measured by inhibiting the tyrosinase activity and cellular melanin production in B16 melanoma cells. Thus, this study could provide information about tobacco seed oil for further development and utilization.

## 2. Results and Discussion

### 2.1. Identification of Chemical Constituents by GC-MS

**Figure 1:**
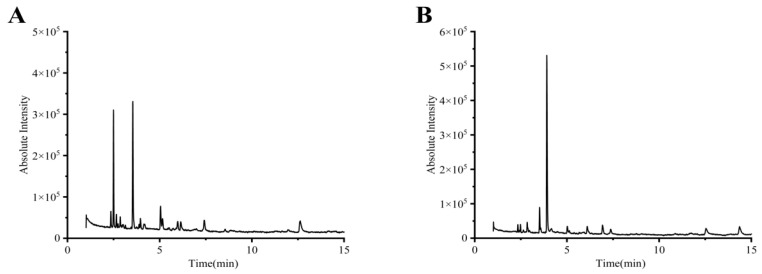
Component diagram of NC89 and BS4 tobacco seed oils. (**A**):NC89; (**B**): BS4.

The information on the chemical composition of tobacco seed oils is limited. The volatile constituents of the NC89 and BS4 tobacco seed oils were analyzed by GC-MS (Figure 1). As shown in Table 1, a total of 10 volatile compounds were characterized by GC-MS in the NC89 tobacco seed oil, including one aromatic hydrocarbon (**6**), one hydrocarbon halides (**2**), two aldehydes (**1**, **3,** and **8**), and four alcohols (**4**, **5**, **7**, and **9**). In the BS4 tobacco seed oil (Table 2), four compounds were detected and identified by the GC-MS.

### 2.2. Composition of Fatty Acid Profiles

The results in Table 3 showed that NC89 and BS4 tobacco seeds are rich in fatty acids, with a content of about 40%. The oil content in these seeds is higher than that of the oils from rape seeds, sunflower seeds, Ricinus communis seeds, and soybeans. A total of 18 different fatty acids were found in these two tobacco seeds oils, in which linoleic acid, oleic acid, and palmitic acid were the major fatty acids, and the contents of these acids in the BS4 were higher than those in the NC89 by the ratios at 71.55%, 14.04%, and 8.83%, respectively. A study has shown that polyunsaturated fatty acids (PUFAs) have important health and nutritional benefits, such as the prevention of cardiovascular disease [11]. Our results revealed that NC89 and BS4 tobacco seed oils are rich in PUFAs, with 74.98% content for the NC89 and 72.84% for the BS4, respectively. These ratios are much higher than the values reported for other important food oils such as olive oils (25%) [12], soybean oils (50.59%) [13], and sesame oils (46%) [14]. The higher unsaturated fatty acid content of tobacco seed oil means that it has higher nutritional value. Tobacco seed oil is widely available in China and has a high economic value, but there are few studies, and this study provides a theoretical basis for the development of tobacco seed oil products.

### 2.3. Ability of Tobacco Seed Oil on Scavenging Free Radicals

**Figure 2:**
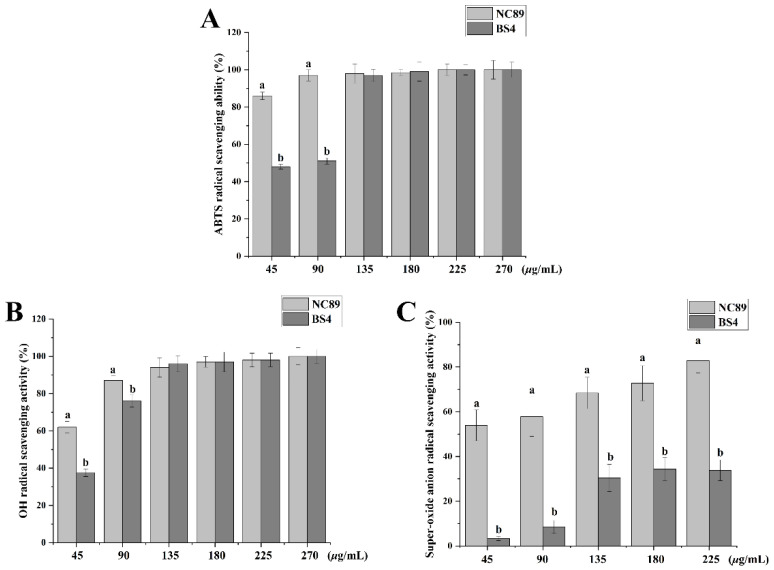
The antioxidant activity of NC89 and BS4 tobacco seed oils. (**A**). ABTS radical scavenging ability, (**B**). OH- radical scavenging activity, (**C**). super-oxide anion radical scavenging activity. Values are presented as the mean ± SD (*n* = 3). The different samll letters above bars present significance of difference (*p* < 0.05).

It is reported that excessive production of free radicals is an important inducer of oxidative stress damage, which is related to the occurrence of some chronic diseases including cancers, and cardiovascular and age-related degenerative diseases [15]. Thereby, how to effectively scavenge free radicals (ABTS, OH^−^, and O_2_^−^) is an important indicator for evaluating the antioxidant ability. As shown in Figure 2, the NC89 and BS4 scavenged the ABTS (Figure 2A), OH^−^ (Figure 2B), and O_2_^−^ (Figure 2C) radicals in a dose-depended manner. Additionally, the NC89 exhibited better antioxidative ability than the BS4 at low concentrations, which may be related to the higher contents of PUFA in the NC89 tobacco seed oil. These results indicate that tobacco seed oils may have a good antioxidative ability. In addition, sunflower oil also exhibited antioxidant activity, in agreement with our results [16].

### 2.4. Inhibitory Effect of Tobacco Seed Oil on Intracellular ROS Generation

The MTT assay results showed that NC89 and BS4 were not toxic to HepG2 cells at a dose of 100 μg/mL. It has been reported that ROS can perform redox signaling on cell signal transduction and oxidative stress, maintaining the human body’s redox equilibrium balance [17]. However, excessive ROS production destroys this balance and leads to abnormal physiological functions and diseases [17]. An H_2_O_2-_induced oxidative stress model in vitro is generally used to explore the antioxidative effect [18]. With the stimulation of H_2_O_2_, the cells produce and secrete excessive ROS to destroy the cell antioxidant defense systems [19]. Therefore, an H_2_O_2_-induced oxidative stress model of HepG2 cells was used in this study to further evaluate the inhibitory effects of these two tobacco seed oils on ROS production.

**Figure 3:**
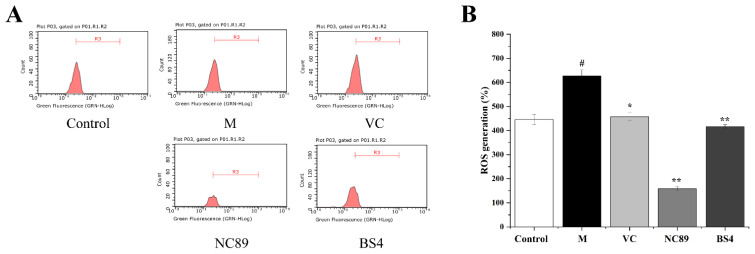
NC89 and BS4 tobacco seeds oil mitigated reactive oxygen species’ (ROS) accumulation in HepG2 cells induced by H_2_O_2_. (**A**): Flow cytometry analysis; (**B**): the intracellular ROS content. Values are presented as the mean ± SD (*n* = 3). ^#^ *p* < 0.05 vs. Control group; * *p* < 0.05, ** *p* < 0.01 vs. M group.

As shown in Figure 3, compared to the control group, the content of H_2_O_2_ was significantly increased in the H_2_O_2_-induced HepG2 cells (*p* < 0.05). Fortunately, the NC89 and BS4 oils remarkedly reduced the ROS accumulation in the H_2_O_2_-induced HepG2 cells (*p* < 0.01). Specifically, it was worth noting that the NC89 had a better capacity of scavenging ROS than the positive control Vc; this may have benefitted from its high contents of PUFA. Our results were supported by another study that showed that the essential oil of *Paederia scandens,* with a high level of PUFA, exhibited antioxidant activity by reducing ROS and MDA levels [20].

### 2.5. Cytoprotective Activity against H_2_O_2_-Induced Cell Apoptosis

**Figure 4:**
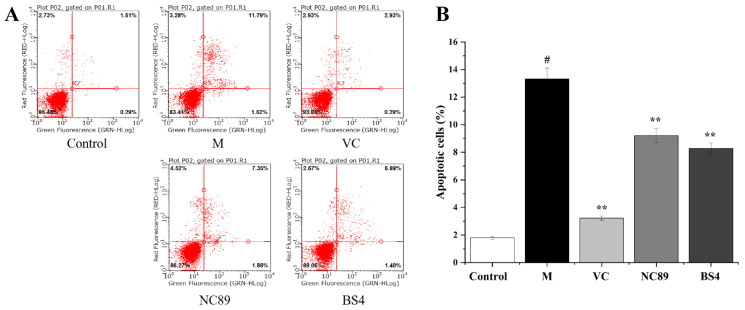
Cytoprotective effects of NC89 and BS4 tobacco seed oils in H_2_O_2_-induced HepG2 cells. (**A**): Flow cytometry analysis; (**B**): the cell apoptosis percentage. Values are presented as the mean ± SD (*n* = 3). ^#^ *p* < 0.05 vs. Control group; ** *p* < 0.01 vs. M group.

Apoptosis is an important procedure for the stable maintenance of the intracellular environment that is regulated by genes [21], while abnormal cell apoptosis induced by oxidative stress and inflammation can cause many diseases, such as cardiovascular disease, neurodegenerative diseases, and atherosclerosis [21]. The cytoprotective activities of tobacco seed oil is shown in Figure 4. In comparison with the control group, the H_2_O_2_ treatment significantly increased the apoptosis rate of the HepG2 cells (*p* < 0.05). Notably, the apoptosis rate was significantly decreased after the NC89 and BS4 oil treatments compared to the model group (*p* < 0.01). The present results were similar to the finding of *nigella sativa* seed oil, which also can inhibit the production of apoptosis [22].

### 2.6. Antioxidant Ability of Tobacco Seed Oil on H_2_O_2_-Induced HepG2 Cells

**Figure 5:**
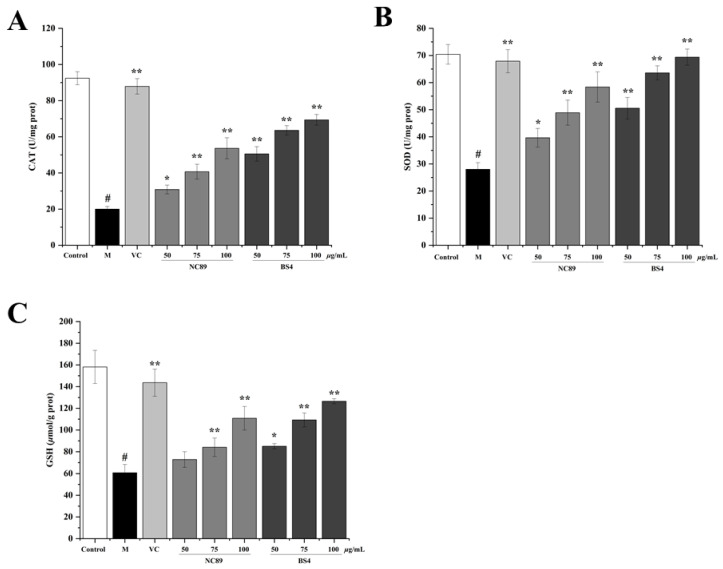
NC89 and BS4 tobacco seed oils enhanced intracellular antioxidant defense enzyme activities in H_2_O_2_-induced HepG2 cells. The content of cellular CAT (**A**), SOD (**B**), and GSH (**C**) were determined by commercially available kit. Values are presented as the mean ± SD (*n* = 3). ^#^ *p* < 0.05 vs. Control group; * *p* < 0.05, ** *p* < 0.01 vs. M group.

SOD and CAT are important endogenous antioxidant enzymes that contribute to the antioxidant defense system in cells to ameliorate the oxidative stress induced by H_2_O_2_ [23]. As the most powerful antioxidant in the body, GSH play a vital role in scavenging free radicals to maintain the intracellular balance of oxidation and reduction. The results of the CAT and SOD activities and the GSH levels are shown in Figure 5. Compared with the control group, the CAT and SOD activities, and the GSH levels were dramatically reduced by the H_2_O_2_ (*p* < 0.01), but significantly increased by the NC89 and BS4 pretreatment in comparison to the model group (*p* < 0.05) in a concentration-dependent manner. The present results reveal that the NC89 and BS4 tobacco seed oils suppressed the oxidative stress by enhancing the intracellular antioxidant defense systems and reducing the ROS accumulation. A previous study indicated that Artemisia scoparia essential oil also increased the activity of antioxidant enzymes, which was consistent with our results [24].

### 2.7. Anti-Inflammatory Effect of Tobacco Seed Oil

**Figure 6:**
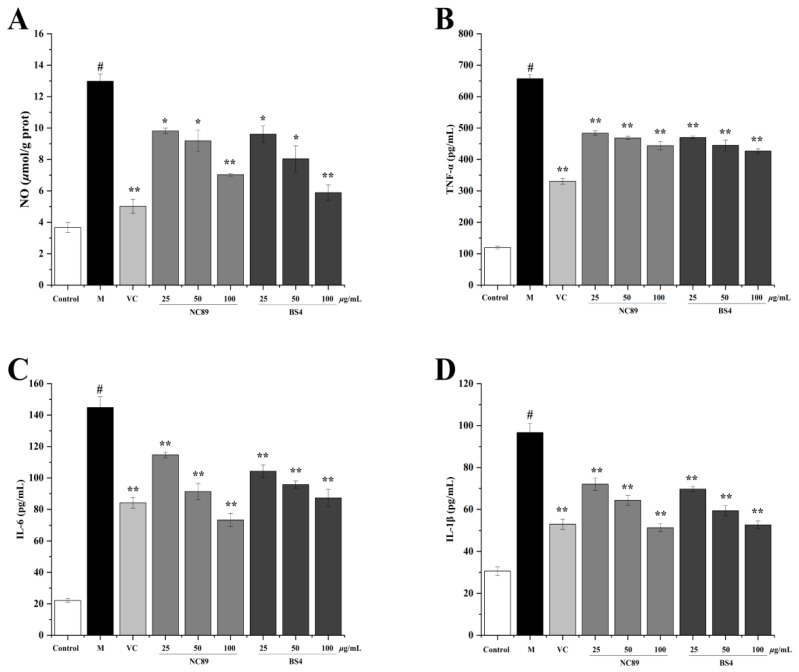
Effects of NC89 and BS4 tobacco seed oils on the secretion of NO, TNF-α, IL-6 and IL-1*β* in LPS-induced RAW264.7 cells. The levels of NO (**A**), TNF-α (**B**), IL-6 (**C**), and IL-1*β* (**D**) were evaluated by kit. Values are presented as the mean ± SD (*n* = 3). ^#^ *p* < 0.05 vs. Control group; * *p* < 0.05, ** *p* < 0.01 vs. M group.

The MTT assay result indicated that NC89 and BS4 were not toxic to RAW 264.7 cells at concentrations less than or equal to 100 μg/mL. In general, inflammation is a disease-fighting response in the body, but a persistent inflammatory response will cause cell damage and even cell apoptosis [25]. It is reported that an overproduction of NO is able to induce an inflammatory response, in which process the inflammatory cytokines’ tumor necrosis factor-α (TNF-α), interleukine-1*β* (IL-1*β*), and interleukine-6 (IL-6) are secreted to exacerbate the inflammatory response [26]. An LPS-induced RAW264.7 cell inflammatory model is widely used to evaluate the anti-inflammatory effect in many studies [27]. As shown in Figure 6, NO content (Figure 6A) and the levels of TNF-α (Figure 6B), IL-1*β* (Figure 6C), and IL-6 (Figure 6D) were dramatically increased by the H_2_O_2_ compared with the control group (*p* < 0.01), but significantly reduced by the NC89 and BS4 (*p* < 0.01) in a dose-dependent manner. It is known that oxidative stress and inflammatory response are mutually reinforcing, and the occurrence and development of many diseases are related to the combined action of oxidative stress and inflammation [26]. These results showed that NC89 and BS4 tobacco seed oils had anti-inflammatory capacities, which were in accordance with the antioxidative results and further supported the results that tobacco seed oils could alleviate cell apoptosis. The present findings were similar to the results of *Zingiber montanum* oil, which also inhibited NO production in LPS-treated RAW264.7 cells, and thus exhibited anti-inflammatory activity [28].

### 2.8. The Inhibitory Effect of Tobacco Seed Oil on the MAPK Pathway

**Figure 7:**
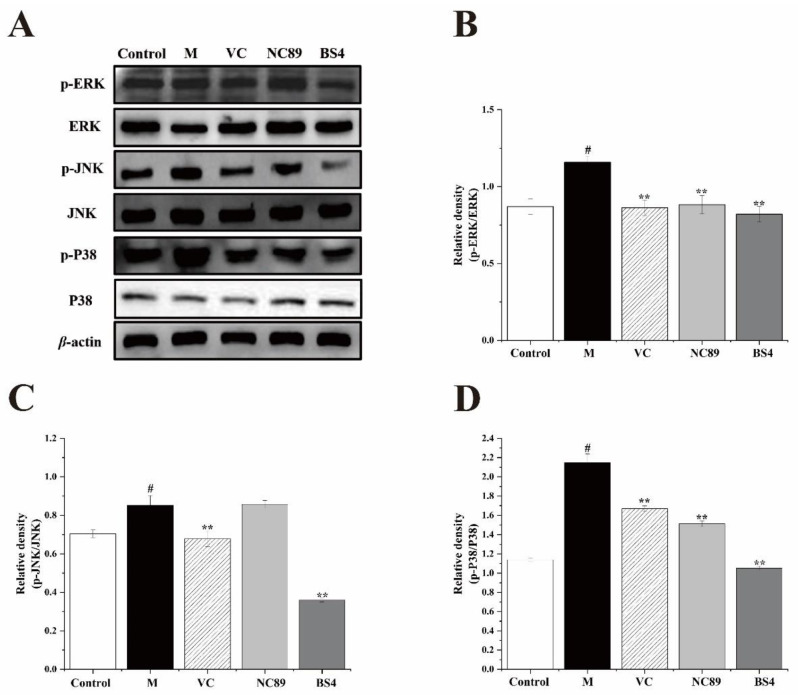
NC89- and BS4-mitigated LPS-induced inflammatory response via the MAPK signal pathway. (**A**) The protein expression of MAPK pathway was determined by Western blotting. (**B**–**D**) The quantification of ERK, JNK, and P38 protein expression was performed by Image J. The values are presented as mean ± SD (*n* = 3). ^#^ *p* < 0.05, vs. the Control group; ** *p* < 0.01, vs. Model group.

The MAPK signaling pathway plays a crucial role in regulating pro-inflammatory cytokines and mediators, which is consistent with ERK, JNK and P38 proteins [29]. Truong previously reported that *Zingiber montanum* oil inhibited the MAPK signaling pathway in LPS-treated RAW 264.7 cells, in agreement with our results [28]. In order to investigate the possible mechanism of tobacco seed oils in LPS-induced RAW264.7 cells, the production of the MAPK pathway-related proteins, including ERK, p-ERK, JNK, p-JNK, P38, and p-p38, were determined by Western blotting analysis. As shown in Figure 7, H_2_O_2_ markedly enhanced the phosphorylation of the ERK (*p* < 0.05), JNK (*p* < 0.05), and P38 (*p* < 0.05) proteins in contrast to the control group. Conversely, the treatment with the NC89 and BS4 tobacco seed oils dramatically reduced the relative ratio of p-ERK/ERK (*p* < 0.01, *p* < 0.01), p-JNK/JNK (*p* > 0.05, *p* < 0.01), and P-p38/P38 (*p* < 0.01, *p* < 0.01) in the LPS-induced RAW264.7 cells. These results precisely demonstrate the reason that tobacco seed oil could effectively inhibit the expression of inflammatory cytokines. Additionally, the MAPK pathway is also strongly associated with oxidative stress, and many studies have reported that oxidative stress can be effectively suppressed via inhibiting the activation of the MAPK pathway [30]. Convincingly, our results further support this view.

### 2.9. Whitening Effect of Tobacco Seed Oils on Inhibition Rate of Tyrosinase Activity

**Figure 8:**
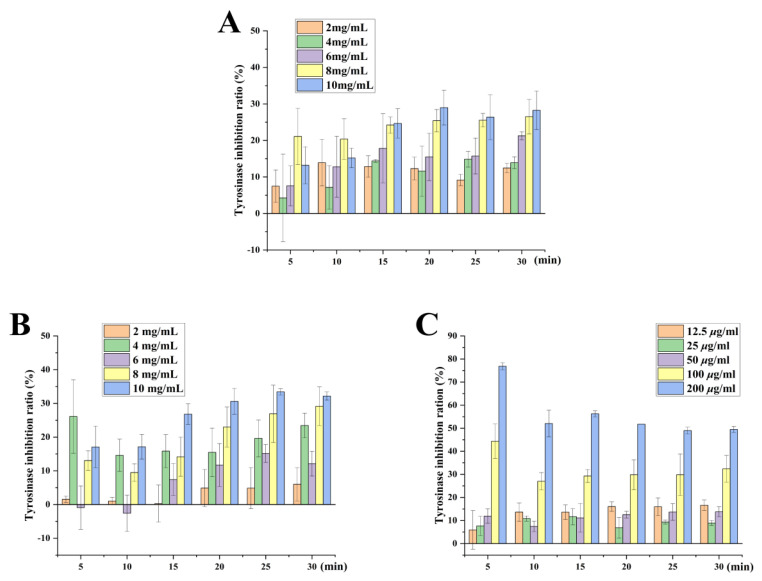
Inhibitory effect of NC89 and BS4 seed oils with different concentrations on tyrosinase in different times. Mushroom tyrosinase was treated with NC89 (**A**), BS4 (**B**), Vitamin C (**C**), and tyrosinase activity was measured. Values are presented as the mean ± SD (*n* = 3).

Tyrosinase is widely distributed in nature and takes part in the first two steps of melanin biosynthesis [31]. Nonetheless, the abnormal accumulation of melanin can trigger skin diseases such as melanoma [32]. Vc is a vital reducing agent that can avoid reactions from dopamine to levodopa by regulating the chemical reduction of dopamine [33] and could suppress melanin production via inhibiting tyrosinase activity [34]. Moreover, a study has shown that saponified evening primrose oil effectively inhibited tyrosinase activity, which is consistent with our result [35]. The whitening effect of NC89 and BS4 was studied by a tyrosinase inhibition experiment. As shown in Figure 8, the inhibitory effects of the NC89 and BS4 on tyrosinase activity increased gradually with time, but their inhibitory effects were obviously inferior to that of Vc. When the concentration was 8 mg/mL, for the NC89, the inhibition of the tyrosinase activity rate was the highest (24.21 ± 2.2%) and tended to be stable for 15 min. At the concentration of 10 mg/mL, the inhibition of tyrosinase activity by the NC89 reached the maximum (28.97 ± 4.7%) at 20 min. For the BS4, the inhibition of tyrosinase activity also showed an upward trend with the increase in time at the concentrations of 8 mg/mL and 10 mg/mL; the inhibition rate of tyrosinase also showed an upward trend with the increase in time. However, when its concentration was 4 mg/mL, the inhibition rate of the tyrosinase reached a maximum (26.12 ± 10.1%) at 5 min and was not significantly different from that of the high-concentration tobacco seed oil. The overall results showed that both tobacco seed oils had strong tyrosinase inhibitory activities, and the BS4 oil was slightly better than the NC89 oil.

### 2.10. Effect of Tobacco Seed Oil on the Proliferation in B16 Cells

As shown in Table 4, arbutin, NC89, and BS4 ranging from 4.2 mg/mL to11.1 mg/mL had no cytotoxicity against the B16 cells in the 24 h and 48 h assessments. However, after 72 h of proliferation, the NC89 and BS4 showed cytotoxicity against the B16 cells when the concentrations of NC89 and BS4 were greater than or equal to 8.3 mg/mL.

### 2.11. Inhibitory Effect of Tobacco Seed Oil on Tyrosinase in B16 Cells

As shown in Table 5, the promotion effect of the NC89 at a concentration of 4.2 mg/mL on the tyrosinase in the B16 cells persisted until 72 h, showing a weak inhibitory effect. With the increase in concentration to 11.1 mg/mL, the NC89 could inhibit the tyrosinase activity in the B16 cells. Similarly, the BS4 (4.2 mg/mL) also promoted tyrosinase production in the B16 cells at 72 h. At 24 h, the BS4 inhibited tyrosinase activity until the concentration was greater than or equal to 6 mg/mL. From 48 h to 72 h, BS4 at a concentration of 4.2 mg/mL inhibited the tyrosinase activity in the B16 cells. With the increases in concentration and time, the inhibitory effects of NC89, BS4, and arbutin on tyrosinase activities increased gradually. The inhibitory effects of NC89 and BS4 (11.1 mg/mL) and arbutin (500 μg/mL) on tyrosinase activities in the B16 cells reached the maximum and the inhibitory rates were 12.91 ± 2.63%, 12.58 ± 1.71%, and 38.1 ± 2.33%, respectively. A previous study showed volatile oil from ginger exhibits a potent inhibitory effect on intracellular tyrosinase activity, which is consistent with our result [36].

### 2.12. Inhibitory Effect of Tobacco Seed Oil on Melanin Production in B16 Cells

Table 6 displays the melanin production in B16 cells at different concentrations of NC89, BS4, and arbutin at 24 h, 48 h, and 72 h. The NC89 inhibited melanin production in the B16 cells when the concentration was greater than or equal to 11.1 mg/mL at 24 h. The BS4 had an inhibitory effect on melanin production in the B16 cells at 24 h when the concentration was greater than or equal to 6 mg/mL. Meanwhile, the inhibitory effects of NC89, BS4, and arbutin on melanin production were positively correlated with the concentration. When the concentrations of NC89 and BS4 were 11.1 mg/mL, their inhibitory effect on melanin formation reached the maximum at 72 h. The inhibition rates of NC89, BS4, and arbutin were 9.78 ± 1.12%, 9.14 ± 2.19%, and 42.82 ± 2.53, respectively. The results in this study show that tobacco seed oil can suppress the production of melanin, showing the potential whitening ability of plant oil. Our finding was also supported by another study, where saponified evening primrose oil effectively reduced melanogenesis in B16 melanoma cells and decreased pigmentation in UV-exposed skin [35].

As a known metabolic enzyme of melanin, tyrosinase can control the activity of melanocytes and determine the rate of melanin synthesis. Therefore, inhibiting tyrosinase activity is an effective way to reduce the synthesis and deposition of melanin. It has been demonstrated that oxidative stress can accelerate the deposition of melanin via promoting the oxidation of dopamine, and an inflammatory response can also affect the synthesis of melanin [9]. In this study, tobacco seed oils exhibited a whitening ability by inhibiting the tyrosinase activity to reduce the melanin deposition, which may be closely related to their antioxidative and anti-inflammatory effects.

## 3. Material and Methods

### 3.1. Chemical and Reagents

Arbutin (purity > 98%), dimethyl sulfoxide (DMSO), V_C_ (purity > 99%), 20,70-dichlorofluorescin diacetate (DCFH-DA), and methylthiazol-2-yl-2,5-diphenyl tetrazolium bromide (MTT) were purchased from Sigma-Aldrich (St. Louis, MO, USA). B16 and HepG2 cells were obtained from the Kunming Cell Bank. Phosphate-buffered saline (PBS), fetal bovine serum (FBS), Dulbecco’s modified Eagle’s medium (DMEM), and penicillin/streptomycin were obtained from Servicebio (Wuhan, China). 1,3,5-tri(2-pyridyl)-2,4,6-triazine (TPTZ), 2,2′-Azino-bis (3-ethylbenzothiazoline-6-sulfonic acid) (ABTS), 2-diphenyl-1-pi-crylhydrazyl radical (DPPH), Folin–Ciocalteu reagent, Trolox, and levodopa were purchased from Sigma-Aldrich (Shanghai, China). Nitric oxide (NO), catalase (CAT), superoxide dismutase (SOD), glutathione peroxidase (GSH), Annexin V−Fluorescein Isothiocyanate (FITC)/Propidium Iodide (PI) cell apoptosis detection kits, and a BCA protein assay kit were purchased from the Beyotime company (Beijing, China). ELISA kits for interleukin (IL)-1*β*, IL-6 and tumor necrosis factor-α (TNF-α) were purchased from MultiSciences (Lianke) Biotech Co. (Hangzhou, China). Tyrosinase and an intracellular tyrosine kinase activity detection kit were obtained from Sigma-Aldrich.

### 3.2. Preparation of Tobacco Seed Oil

Tobacco seeds of the NC89 and BS4 species were collected from the seed breeding base of Yuxi Zhongyan Tobacco Seed Co., Ltd., in 2019. The cold-processed method was used to extract the tobacco seed oil. In brief, at room temperature, the tobacco seed oil was directly pressed using a small oil press machine and then centrifuged at 1000× *g* for 10 min. The oil was carefully collected for further research.

### 3.3. Chemical Composition of Tobacco Seed Oil Using GC/MS Analysis

The volatile components in the tobacco seed oil were extracted by the headspace solid-phase microextraction (HS-SPME) method [37]. Then, the chemical compositions of the NC89 and BS4 tobacco seed oils were analyzed by GC-MS equipment (GC-2010 Plus, Shimadzu, Kyoto, Japan). Briefly, 3 mL of tobacco seed oil was added to a 20 mL screw-capped amber glass vial and immediately sealed. The extraction was performed by SPME fiber composed of 50/30 µm DVB/CAR/PDMS at 40 °C for 20 min. The extract was inserted in the GC injection port immediately for desorption (3 min, 240 °C). High-purity helium (>99.999%) was used as the carrier gas, and the column was an HP-5 quartz capillary column (30 m × 0.32 mm, 0.25 µm). The condition was as follows: 0–40 min (40–80 °C), 40–47.5 min (80–250 °C), and 47.5–57.5 min (250 °C). The mass data was obtained at a scan range of 35–500 m/z in an electron ionization mode at 70 eV. The identification of volatile compounds was performed by comparison with the reported data in the NIST 2014 library.

### 3.4. Determination of Fatty Acid Composition in Tobacco Seed Oil

The fatty acid composition was analyzed according to a previously reported method [38]. First, the fatty acids of the tobacco seed oil were transformed into fatty acid methyl esters, which were further analyzed using GC equipment, coupled with an autoinjector (Santa Clara, CA, USA, Agilent, 7890A) and a flame ionization detector (FID). The chromatography column was BD-23 (0.25 μm, 60 m × 0.25 mm, USA, Agilent). The oven condition was as follows: 0–3 min (100 °C), 3–6.5 min (100–170 °C), 6.5–16.5 min (170 °C), 16.5–19.5 (170–200 °C), 19.5–24.5 (200 °C), 24.5–39.5 (200–230 °C), and 39.5–44.5 (230 °C). The flow rate of the high-purity helium was 2.0 mL/min, with a split ratio of 1:29.5. The injector temperature was set at 270 °C, and the detector temperature was set at 280 °C, respectively. The identification was performed by comparison with the standard compounds. The quantification was carried out, and the data was expressed as mg/g oil.

### 3.5. Antioxidant Activity Assessment

#### 3.5.1. Scavenging Effect on ABTS Radicals

The ABTS scavenging effect was carried out using an improved method [18]. Briefly, ABTS+ (7 mM) was added to a phosphoric acid buffer (2.5 mM) for 16 h at room temperature in the dark to obtain the ABTS solution. The ABTS solution was diluted with methanol to adjust its absorbance to 0.70 ± 0.02 at 734 nm. The tobacco seed oil (45, 90, 135, 180, 225, and 270 μg/mL) were mixed with the ABTS solution (total system, 300 µL) for a 30 min incubation in the dark at 37 °C. Distilled water was used as a control. The absorbance was recorded at 734 nm. The ABTS radical scavenging activity was calculated using the following formula:

[(A_control_ − A_sample_/A_control_] × 100% (1)

#### 3.5.2. Scavenging Effects on OH^-^ Radicals

The scavenging effect on the OH- free radicals was estimated according to a reported method [39]. The stock solutions for the Fenton’s reaction were prepared at the following ratio: 0.02 M FeSO_4_ to 0.01 M salicylic acid to 0.02 M H_2_O_2_ = 1:2:1. The H_2_O_2_ was replaced with distilled water as a control. The tobacco seed oil in different concentrations (45, 90, 135, 180, 225, and 270 μg/mL) was mixed with Fenton’s reaction solution (total volume, 300 µL) for a 30 min reaction. The absorbance was determined at 536 nm. The OH^−^ radical scavenging activity was calculated using the following formula:

[A_control_ − (A_sample_ − A_sample_ control)]/A_control_ × 100% (2)

#### 3.5.3. Scavenging Effects on O_2_^−^ Radicals

The scavenging effect on the O_2_^−^ free radicals was estimated according to a reported method [40]. The reagents (A) and (B) were prepared at the following ratios: 5 mM pyrogallic acid to Tris-HCl buffer (1:7), and 5 mM pyrogallic acid to 10 nM HCl buffer (1:7), respectively. The samples (45, 90, 135, 180 and 225 μg/mL) were mixed with a reaction solution (total volumes, 300 µL) to test the O_2_^-^ radical scavenging ability of the tobacco seed oil. The reagents (A) and (B), respectively, were added to the sample and control groups. The oil was replaced with distilled water as a control by adding the reagent (A). The absorbance was measured at 325 nm after 5 min of reaction. The O_2_^−^ radical scavenging activity was calculated using Formula (2).

### 3.6. Cytoprotective Effect on H_2_O_2_-Induced HepG2 Cells

#### 3.6.1. HepG2 Cell Culture and Cell Viability Assay

HepG2 cells were cultured in DMEM medium with 10% FBS in a cell incubator with 5% CO_2_ at 37 °C [41]. The cells’ viability after the treatment with the NC89 and BS4 tobacco seed oils (12.5, 25, 50, 100, and 200 μg/mL) was assessed using an MTT (methylthiazol-2-yl-2,5-diphenyl tetrazolium bromide) assay. In brief, the HepG2 cells (1.0 × 105 cells/well) were seeded in 96-well plates for 24 h. The incubation of the tobacco seed oils at different concentrations with the HepG2 cells were maintained for 20 h. Afterward, 0.5 mg/mL MTT solution was added. After 4 h incubation, the MTT solution was discarded, and 200 µL DMSO was added to each well in order to solubilize the purple formazan crystals. The absorbance was recorded at 490 nm.

#### 3.6.2. Inhibitory Effects on the ROS Generation

HepG2 cells were seeded in a 12-well plate (1 × 10^5^ cells/well) in an incubator at 37 °C with 5% CO_2_ for 12 h. The cells were pretreated with tobacco seed oils at a concentration of 100 μg/mL for 24 h. Then, the cells were induced for another 24 h with 1 mM H_2_O_2_. After washing with PBS, the cells were mixed with 1 mL DCFH-DA solution (10 μM) for a 30 min incubation. After washing twice with pre-cooled PBS, the fluorescence intensity was measured using flow cytometry.

#### 3.6.3. Inhibitory Effects on Cell Apoptosis

The cell apoptosis of the HepG2 cells was measured using an Annexin V-FITC/PI apoptosis kit. HepG2 cells at a density of 1 × 10^5^ cells per well were seeded in 6-well plates and treated with tobacco seed oil (100 μg/mL) for 24 h. Then, the cells were treated with 1 mM H_2_O_2_ for another 6 h. Finally, the cell apoptosis was determined by Annexin V/FITC and PI staining using a flow cytometer.

#### 3.6.4. Analysis of Intracellular SOD, CAT Activities and GSH Content

To analyze the influence of tobacco seed oil on intracellular enzyme activities, the HepG2 cells after the tobacco seed oil treatment were collected and homogenized in a phosphate buffer. The intracellular CAT and SOD activities and the GSH content were determined using commercial kits according to the instruments.

### 3.7. Anti-Inflammatory Activity of Tobacco Seed Oil

#### 3.7.1. Determination of NO, IL-1β, IL-6, and TNF-α Levels in RAW264.7 Cells

The cell culture of RAW264.7 cells and the cell viability assay were performed as described in Section 3.6.1. Both the NC89 and BS4 tobacco seed oils had no cytotoxicity against RAW264.7 cells at 100 μg/mL. Then, the RAW264.7 cells (1 × 105 cells/well) were seeded in 24-well plates for 24 h, and then cultured in a medium containing tobacco seed oil at a concentration of 100 μg/mL for 4 h. Except for the control group, the cells in the tobacco seed oils groups were treated with lipopolysaccharide (LPS, 1.0 μg/mL) for 20 h [42]. After centrifugation at 1500× *g* for 10 min, the cell medium was collected. The productions of NO, TNF-α, IL-1*β*, and IL-6 were evaluated using commercial assay kits (MultiSciences Biotech, Hangzhou, China).

#### 3.7.2. Determination of MAPK Signaling Pathway Proteins

The proteins in the MAPK signaling pathway were measured by Western blotting analysis [43]. Briefly, a lysis buffer containing 1% protease inhibitor and 10% phosphatase inhibitor was used to extract the cell proteins. The protein concentration was determined by a BCA protein assay kit. The proteins were isolated on SDS-PAGE gels and transferred to polypropylene fluoride (PVDF) membranes. After incubation with primary antibodies, the bound proteins were incubated with the corresponding secondary antibodies. Finally, the quantification of ERK, p-ERK, JNK, p-JNK, P38, and p-p38 proteins in the MAPK pathway was determined by an enhanced chemiluminescent detection reagent.

### 3.8. Whitening Effect of Tobacco Seed Oils

#### 3.8.1. Inhibitory Effect on Tyrosinase Activity

The NC89 and BS4 tobacco seed oils were prepared using butylene glycol-PBS solution with concentrations of 2.0, 4.0, 6.0, 8.0, and 10.0 mg/mL according to the method described in a previous study [44]. Vc (12.5, 25, 50, 100, and 200 μg/mL) was used as a positive control. Then, 40 μL of sample solution, 40 μL of substrate solution, and 100 μL of PBS was mixed for a 10 min incubation. After that, 20 μL of tyrosinase was added. After 5.0, 10.0, 15.0, 20.0, 25.0, and 30.0 min of reaction, the absorbance values were measured. The groups were as follows: (1) sample group: sample + substrate + PBS + tyrosinase; (2) control group: sample + PBS + tyrosinase; (3) blank group: substrate + PBS + tyrosinase; (4) control group: PBS + tyrosinase. The inhibitory effect on the tyrosinase activity was calculated as follow:

[(A_blank_ − A_control_) − (A_sample_ − A_sample control_]/(A_blank_ − A_control_) × 100% (3)

#### 3.8.2. The Cytotoxicity of Tobacco Seed Oils on B16 Melanoma Cells

The cell culture and cell viability assay of the B16 cells were performed as described in Section 3.6.1. The B16 melanoma cells were inoculated in 96-well plates at 1 × 10^5^ cells/mL and cultured in DMEM containing 10% FBS and 1% dual antibiotics in a cell incubator with 5% CO_2_. The medium was discarded after 24 h. The cells were then treated with the tobacco seed oils (4.2, 5, 6, 8.3, and 11.1 mg/mL) and arbutin (31, 62.5, 125, 250, 250, and 500 μg/mL), respectively. In the control group, the cells were treated with equal volumes of the medium. After incubation for 24, 48, and 72 h, respectively, the MTT solution was added to treat the cells for 4 h. Then, DMSO was added to each well, and they were shaken for 10 min. The absorbance was measured at 490 nm.

#### 3.8.3. Inhibitory Effect on Tyrosinase Level in B16 Melanoma Cells

B16 melanoma cells (1 × 10^5^ cells/mL) were inoculated in 96-well plates. The medium was discarded after 24 h [45]. Arbutin was used as a positive control. The cells were then treated with a medium containing tobacco seed oil or arbutin. After 24, 48, and 72 h incubation, respectively, the medium was discarded. The cells were washed three times with PBS, added to 150 μL of 10 % TritonX-100 solution, and then placed in refrigeration at −80 °C for 1 h. After thawing at room temperature, the cells were incubated at 37 °C and reacted with 40 μL of 2 mM levodopa solution for 1 h. Finally, the absorbance value was measured at 475 nm.

#### 3.8.4. Inhibitory Effects on Melanin Synthesis

B16 melanoma cells were inoculated into 12-well plates at 1 × 10^5^ cells/mL [46]. The cell culture of the B16 cells were performed as described in Section 3.8.2. After the treatment with the tobacco seed oil or arbutin, the cells were collected. Then, 1 mL of 1 M NaOH solution (containing 10% DMSO) was added in a water bath for 2 h at 80 °C. After the cells were completely dissolved and broken down, the supernatant (200 μL) was transferred to a 96-well plate for 5 min, and the absorbance values were detected at 405 nm.

### 3.9. Statistical Analysis

The study data were expressed as the means ± standard deviation (SD). The quantification was carried out using image analysis software (ImageJ, 1.46a; NIH, Bethesda, MD, USA). The significant differences were analyzed by one-way ANOVA and Tukey’s test using Origin 8.5 software. A *p*-value less than 0.05 (*p* < 0.05) was considered statistically significant.

## 4. Conclusions

The NC89 and BS4 tobacco seed oils have a high fatty acid content, especially PUFAs. Linoleic acid was the most abundant PUFA in both the NC89 and BS4 oils. The NC89 and BS4 had strong scavenging capacities for ABTS, OH^-^, and O_2_^−^ radicals and significantly inhibited ROS production in H_2_O_2_-induced in HepG2 cells. The possible cytoprotective effects of NC89 and BS4 tobacco seed oils might be closely related to the regulation of antioxidative enzyme activities and cell apoptosis. In addition, they had significant anti-inflammatory activities by inhibiting the production of pro-inflammatory cytokines, including TNF-α, IL-1*β*, and IL-6, via the MAPK signaling pathway. In addition, NC89 and BS4 showed a whitening activity by inhibiting tyrosinase activity and cellular melanin production. In conclusion, the research on the nutritional properties and health benefits of tobacco seed oils provides the knowledge that tobacco seed oils can be used as a valuable oil resource in food and cosmetic applications.

## Figures and Tables

**Table 1:** Volatile constituents of NC89 tobacco seed oil.

Peak	t_R_ (min)	CAS	Molecular Formula	Molecular Weight	Compounds
1	2.355	32749-94-3	C_7_H_14_O	114.19	2,3-Dimethylpentanal
2	2.655	107-84-6	C_5_H_11_Cl	106.59	Chloroisopentane
3	2.870	58735-67-4	C_8_H_16_O	128.212	2-Ethyl-hexanal
4	5.040	123-51-3	C_5_H_12_O	88.15	3-Methyl-1-butanol
5	5.165	15250-22-3	C_10_H_22_O	158.2811	2,7-Dimethyl-1-octanol
6	5.985	108-88-3	C_7_H_8_	92.14	Toluene
7	6.155	71-41-0	C_5_H_12_O	88.15	1-Pentanol
8	7.420	66-25-1	C_6_H_12_O	100.16	Hexanal
9	12.62	111-27-3	C_6_H_14_O	102.17	Hexyl alcohol

**Table 2:** Volatile constituents of BS4 tobacco seed oil.

Peak	t_R_ (min)	CAS	Molecular Formula	Molecular Weight	Compounds
1	2.340	16630-91-4	C_8_H_16_O	128.212	2-methyl heptanal
2	2.845	3010-96-6	C_8_H_16_O_2_	144.2114	2,2,4,4-Tetramethyl 1,3-cyclobutanediol
3	3.910	543-75-9	C_4_H_6_O_2_	86.0892	2,3-dihydro-1,4-Dioxin
4	14.350	110-43-0	C_7_H_14_O	114.19	2-Heptanone

**Table 3:** Composition and relative contents of tobacco seed oil fatty acids (%).

Fatty Acids	NC89	BS4
butyric acid	0.32	0.14
myristic acid	0.03	0.03
palmitic acid	8.18	8.83
palmitoleic acid	0.10	0.12
heptadecanoic acid	0.13	0.12
10-heptadecenoic acid	0.06	0.06
stearic acid	3.21	3.30
oleic acid	12.45	14.04
linolelaidic	0.42	0.39
linoleic acid	73.53	71.55
α-linoleic acid	0.93	0.82
arachidic acid	0.21	0.21
eicosenoic acid	0.13	0.13
11,14-eicosadienoic acid	0.10	0.08
heneicosanoic acid	0.02	0.01
behenic acid	0.08	0.10
carnaubic acid	0.08	0.05
tetracosenic acid	0.05	0.03
SFA	12.24	12.79
MUFA	12.78	14.37
PUFA	74.98	72.84

SFA: saturated fatty acids. MUFA: monounsaturated fatty acids. PUFA: polyunsaturated fatty acids.

**Table 4:** Effect of tobacco seed oil on the proliferation in B16 cells.

Sample	Concentration	Viability (%)		
		24 h	48 h	72 h
NC89	4.2 mg/mL	110.18 ± 1.72 *	104.14 ± 4.06	114.95 ± 4.96
BS4	4.2 mg/mL	105.43 ± 6.96 *	112.24 ± 3.5	108.8 ± 4.78
Arbutin	31 μg/mL	124.20 ± 3.84	96.9 ± 2.41	90.38 ± 3.93
NC89	5 mg/mL	105.43 ± 2.66 *	102.91 ± 1.83	106.25 ± 2.77
BS4	5 mg/mL	107.93 ± 3.86	106.02 ± 2.02	108.38 ± 3.44
Arbutin	62.5 μg/mL	113.15 ± 6.23	93.54 ± 3.27	88.98 ± 7.35
NC89	6 mg/mL	108.58 ± 5.06 *	102.2 ± 2.48	110.08 ± 2.88
BS4	6 mg/mL	117.07 ± 6.7	113.22 ± 1.83	95.15 ± 3.98
Arbutin	125 μg/mL	124.77 ± 3.71	92.8 ± 1.22	92.31 ± 1.26
NC89	8.3 mg/mL	119.00 ± 2.32	103.34 ± 1.81	94.12 ± 1.95
BS4	8.3 mg/mL	125.07 ± 3.57	106.59 ± 2.51	84.43 ± 2.83 *
Arbutin	250 μg/mL	112.22 ± 6.27	92.41 ± 0.51	93.11 ± 2.32
NC89	11.1 mg/mL	126.32 ± 3.65	90.15 ± 1.52	89.73 ± 2.63
BS4	11.1 mg/mL	127.25 ± 1.71	91.78 ± 2.25	81.1 ± 3.08 *
Arbutin	500 μg/mL	107.36 ± 3.23	90.38 ± 1.37	93.12 ± 2.16

Values are presented as the mean ± SD (*n* = 3). * *p* < 0.05 vs. arbutin group.

**Table 5:** Inhibitory effect of tobacco seed oil on tyrosinase in B16 cells.

Sample	Concentration	Inhibition Ratio (%)		
		24 h	48 h	72 h
NC89	4.2 mg/mL	−5.58 ± 4.46	−1.79 ± 2.05	0.47 ± 1.26
BS4	4.2 mg/mL	−5.58 ± 2.41	−4.77 ± 2.67	−1.04 ± 2.58
Arbutin	31 μg/mL	−5.63 ± 2.39	3.19 ± 1.12	9.41 ± 3.19
NC89	5 mg/mL	−3.7 ± 4.28	−0.05 ± 1.91	2.32 ± 1.8
BS4	5 mg/mL	−1.82 ± 4.19	0.42 ± 2.92	1.48 ± 1.54
Arbutin	62.5 μg/mL	0.58 ± 1.86	6.75 ± 2.02	15.27 ± 3.22
NC89	6 mg/mL	−1.5 ± 1.98	1.19 ± 2.28	4.17 ± 1.81
BS4	6 mg/mL	1.94 ± 2.41	3.25 ± 3.35	4.34 ± 1.46
Arbutin	125 μg/mL	1.82 ± 1.66	12.53 ± 2.81	22.88 ± 2.49
NC89	8.3 mg/mL	−1.19 ± 3.69	6.25 ± 3.14	8.88 ± 3.21
BS4	8.3 mg/mL	5.39 ± 2.26	6.65 ± 3.06	7.87 ± 1.84
Arbutin	250 μg/mL	9.27 ± 2.81	24.09 ± 3.5	28.26 ± 2.8
NC89	11.1 mg/mL	4.76 ± 3.65	10.13 ± 2.28	12.91 ± 2.63
BS4	11.1 mg/mL	7.89 ± 1.88	10.63 ± 1.57	12.58 ± 1.71
Arbutin	500 μg/mL	15.17 ± 1.25	33.87 ± 1.06	38.1 ± 2.33

Values are presented as the mean ± SD (*n* = 3).

**Table 6:** Inhibitory effect of tobacco seed oil on melanin production in B16 cells.

Sample	Concentration	Inhibition Ratio (%)		
		24 h	48 h	72 h
NC89	4.2 mg/mL	−8.3 ± 2.13	1.91 ± 1.45	2.14 ± 1.82
BS4	4.2 mg/mL	−3.15 ± 0.84	1.5 ± 2.83	1.43 ± 2.76
Arbutin	31 μg/mL	5.85 ± 1.08	5.64 ± 1.94	3.23 ± 1.67
NC89	5 mg/mL	−5.55 ± 3.4	4.12 ± 1.22	3.69 ± 2.28
BS4	5 mg/mL	−1.95 ± 1.7	3.76 ± 2.85	3.93 ± 0.82
Arbutin	62.5 μg/mL	6.95 ± 1.59	14.36 ± 2.6	10.05 ± 2.91
NC89	6 mg/mL	−3.67 ± 2.04	4.93 ± 1.88	6.33 ± 3.6
BS4	6 mg/mL	1.88 ± 1.31	5.52 ± 3.32	4.78 ± 3.06
Arbutin	125 μg/mL	14.59 ± 1.44	26.81 ± 1.61	24.99 ± 1.24
NC89	8.3 mg/mL	−0.72 ± 1.14	5.8 ± 1.08	7.05 ± 3.41
BS4	8.3 mg/mL	3.25 ± 1.08	5.21 ± 1.08	5.88 ± 1.38
Arbutin	250 μg/mL	23.25 ± 0.43	35.09 ± 1.23	37.43 ± 1.85
NC89	11.1 mg/mL	6.31 ± 1.86	6.42 ± 2.33	9.78 ± 1.12
BS4	11.1 mg/mL	5.63 ± 1.06	7.81 ± 2.99	9.14 ± 2.19
Arbutin	500 μg/mL	35.49 ± 1.25	39.71 ± 1.66	42.82 ± 2.53

Values are presented as the mean ± SD (*n* = 3).

## Data Availability

The data presented in this study are available on request from the corresponding author.
